# Genome mining as a biotechnological tool for the discovery of novel biosynthetic genes in lichens

**DOI:** 10.3389/ffunb.2022.993171

**Published:** 2022-10-03

**Authors:** Garima Singh, Francesco Dal Grande, Imke Schmitt

**Affiliations:** ^1^ Senckenberg Biodiversity and Climate Research Centre (SBiK-F), Frankfurt am Main, Germany; ^2^ LOEWE Center for Translational Biodiversity Genomics (TBG), Frankfurt am Main, Germany; ^3^ Department of Biology, University of Padova, Padova, Italy; ^4^ Institute of Ecology, Diversity and Evolution, Goethe University, Frankfurt am Main, Germany

**Keywords:** secondary metabolites, Big-FAM, natural products, drug discovery, BiG-SLiCE, medicinal fungi, antiSMASH, MIBiG

## Abstract

Natural products (NPs) and their derivatives are a major contributor to modern medicine. Historically, microorganisms such as bacteria and fungi have been instrumental in generating drugs and lead compounds because of the ease of culturing and genetically manipulating them. However, the ever-increasing demand for novel drugs highlights the need to bioprospect previously unexplored taxa for their biosynthetic potential. Next-generation sequencing technologies have expanded the range of organisms that can be explored for their biosynthetic content, as these technologies can provide a glimpse of an organism’s entire biosynthetic landscape, without the need for cultivation. The entirety of biosynthetic genes can be compared to the genes of known function to identify the gene clusters potentially coding for novel products. In this study, we mine the genomes of nine lichen-forming fungal species of the genus *Umbilicaria* for biosynthetic genes, and categorize the biosynthetic gene clusters (BGCs) as “associated product structurally known” or “associated product putatively novel”. Although lichen-forming fungi have been suggested to be a rich source of NPs, it is not known how their biosynthetic diversity compares to that of bacteria and non-lichenized fungi. We found that 25%–30% of biosynthetic genes are divergent as compared to the global database of BGCs, which comprises 1,200,000 characterized biosynthetic genes from plants, bacteria, and fungi. Out of 217 BGCs, 43 were highly divergant suggesting that they potentially encode structurally and functionally novel NPs. Clusters encoding the putatively novel metabolic diversity comprise polyketide synthases (30), non-ribosomal peptide synthetases (12), and terpenes (1). Our study emphasizes the utility of genomic data in bioprospecting microorganisms for their biosynthetic potential and in advancing the industrial application of unexplored taxa. We highlight the untapped structural metabolic diversity encoded in the lichenized fungal genomes. To the best of our knowledge, this is the first investigation identifying genes coding for NPs with potentially novel properties in lichenized fungi.

## Introduction

Natural products (NPs) are structurally diverse molecules that are produced by nearly all organisms, including plants, fungi, and bacteria. Historically, NPs have played a key role in drug discovery owing to their broad range of pharmacological effects, encompassing antimicrobial, antitumor, and anti-inflammatory properties and protection against cardiovascular diseases (Newman and Cragg, 2012; Newman and Cragg, 2020). In recent decades, about 70% of new drugs have been developed from NPs or NP analogs (Newman and Cragg, 2012; Newman and Cragg, 2020). The demand for novel drugs, however, is ever increasing because of the emergence of antibiotic-resistant pathogens and new diseases, the existence of diseases for which no efficient treatments are available yet, and the need for current drugs to be replaced due to the toxicity or side-effects associated with their use (Demain, 2014; Chakraborty et al., 2021). One way to address global health threats and accelerate NP-based drug discovery efforts is to bioprospect unexplored taxa to assess their biosynthetic potential and to identify potentially novel drug leads.

The genes involved in the synthesis of NPs are often grouped together in biosynthetic gene clusters (BGCs) (Jensen, 2016; Calcott et al., 2018; Keller, 2019). BGCs typically have a core gene that codes for the backbone structure of the NP, and other genes that may be involved in the modification of the backbone or may have regulatory or transport-related functions (Aigle et al., 2014; Rigali et al., 2018; Keller, 2019; Kim et al., 2021). Depending on the core gene, BGCs are grouped into the following major classes: non-ribosomal peptide synthetases (NRPSs), polyketide synthases (PKSs), hybrid non-ribosomal peptide synthetase–polyketide synthases (NRPS–PKSs), terpenes, and ribosomally synthesized and post-translationally modified peptides (RiPPs). The conserved motifs of the core genes facilitate the bioinformatic detection of the clusters (Medema et al., 2011; Bertrand et al., 2018; Calchera et al., 2019; Kum and İnce, 2021).

Traditionally, a large proportion of NP-based drugs have been contributed by a few organisms, as drug discovery has mostly been restricted to culturable organisms (Newman et al., 2003; Cragg and Newman, 2013; Yuan et al., 2016). In recent decades, the bioinformatic prediction of biosynthetic genes or BGCs (i.e., groups of two or more genes that are clustered together and are involved in the production of a secondary metabolite) has revolutionized NP-based drug discovery. This process is culture independent, and enables rapid identification of the entire biosynthetic landscape, including silent or unexpressed genes, from so far unexplored NP resources. Two tools have been vital to the bioinformatic approach to drug discovery: antiSMASH (Blin et al., 2019) and Minimum Information about a Biosynthetic Gene cluster (MIBiG) (Kautsar et al., 2020). antiSMASH includes one of the largest databases for BGC prediction (Blin et al., 2019), whereas MIBiG is a data repository that allows functional interpretation of target BGCs by comparison with BGCs with known functions (Kautsar et al., 2020). Recently, efforts have been made to cluster homologous BGCs into gene cluster families (GCFs) and to simultaneously identify novel BGCs (Kautsar et al., 2021a; Kautsar et al., 2021b). Two tools have been introduced to cluster BGCs into GCFs. BiG-FAM clusters structurally and functionally related BGCs into GCFs, and structurally identify the most diverse BGCs by comparing the query BGCs with about 1,200,000 BGCs in the BiG-FAM database (Kautsar et al., 2021a). BiG-SLiCE clusters homologous BGCs of a dataset into GCFs, without reference to an external database, to identify the unique BGCs in it (Kautsar et al., 2021b). Bioinformatic prediction and clustering of BGCs allow rapid identification of potentially novel drug leads, reducing the cost and time associated with drug discovery by early elimination of unpromising candidates.

Lichens are symbiotic organisms composed of fungal and photosynthetic partners (green algae or cyanobacteria, or both). It has been suggested that they are potentially rich sources of biosynthetic genes and NPs (Boustie and Grube, 2005; Shukla et al., 2010; Shrestha and St. Clair, 2013). Although the number of identified NPs per lichen-forming fungus (LFF) is typically fewer than five (Lumbsch, 1998), the number of BGCs in the genomes of LFF may range from 25 to 60 (Calchera et al., 2019). It is not known how BGCs from LFF relate in structure and function to BGCs from bacteria and non-lichenized fungi (i.e., if a portion of the BGC landscape of LFF is distinct, and might serve as a source of NPs with novel therapeutic properties). Difficulties associated with the heterologous expression of LFF genes have so far restricted the application of LFF-derived NPs in the industry. Recently, two biosynthetic genes from LFF have been successfully heterologously expressed (Kealey et al., 2021; Kim et al., 2021). This, combined with advances in long-read sequencing technology, high quality genomes, and the low cost of sequencing, provides a promising way forward to discover LFF-derived NPs with novel pharmacological potential.

Here we mine and compare the long-read sequencing derived genomes of nine species of the lichenized fungal genus *Umbilicaria* to estimate the functional diversity of BGCs present in them. Specifically, we aim to answer the following questions: (1) what is the functional diversity of BGCs in *Umbilicaria*? and (2) what is the percentage of novel BGCs and species-specific BGCs in *Umbilicaria*?

## Materials and methods

### Dataset

The genomes of the following *Umbilicaria* species were used for this study: *U. deusta*, *U. freyi*, *U. grisea*, *U. subpolyphylla*, *U. hispanica*, *U. phaea*, *U. pustulata*, *U. muhlenbergii*, and *U. spodochroa*. Apart from *U. muhlenbergii*, which belongs to the BioProject PRJNA239196, all the other genomes are a part of BioProject PRJNA820300 (**Table 1**). The details of sample and genomic library preparation, as well as genome sequencing, for *U. muhlenbergii* are available in Park et al. (2014) and for the other eight *Umbilicaria* spp. in Singh et al. (2022). Briefly, all the genomes except *U. muhlenbergii* were generated *via* PacBio SMRT sequencing on the Sequel System II (Radboud University Medical Center (Radboudumc) in Nijmegen, the Netherlands) using the continuous long-read (CLR) mode or the circular consensus sequencing mode. The CLR reads were then processed into highly accurate consensus sequences (i.e., HiFi reads) and assembled into contigs using the assembler metaFlye v2.7 (Kolmogorov et al., 2019). The contigs were then scaffolded with Long Reads Scaffolder (LRScaf) v1.1.12 (github.com/shingocat/lrscaf; Qin et al., 2019). We used only binned Ascomycota reads for this study [extracted using blastx in DIAMOND (more-sensitive, frameshift 15, range-culling) on a custom database and following the MEGAN6 Community Edition pipeline (Huson et al., 2016).

**Table 1:** Voucher information of the genomes used in the study.

Organism	Sample ID	Sequencing technology	BioProject	BioSample	Genome accession no.
*U. deusta*	TBG_2334	PacBio Sequel II	PRJNA820300	SAMN26992774	JALILR000000000
*U. freyi*	TBG_2329	PacBio Sequel II	PRJNA820300	SAMN26992773	JALILQ000000000
*U. grisea*	TBG_2336	PacBio Sequel II	PRJNA820300	SAMN26992780	JALILX000000000
*U. hispanica*	TBG_2337	PacBio Sequel II	PRJNA820300	SAMN26992775	JALILS000000000
*U. muhlenbergii*	KoLRI No. LF000956	Illumina HiSeq	PRJNA239196	SAMN02650300	GCA_000611775.1
*U. phaea*	TBG_1112	PacBio Sequel II	PRJNA820300	SAMN26992776	JALILT000000000
*U. pustulata*	TBG_2345	PacBio Sequel II	PRJNA820300	SAMN26992777	JALILU000000000
*U. spodochroa*	TBG_2434	PacBio Sequel II	PRJNA820300	SAMN26992778	JALILV000000000
*U. subpolyphylla*	TBG_2324	PacBio Sequel II	PRJNA820300	SAMN26992779	JALILW000000000

Two metrics were used to evaluate the quality of the genomes: completeness and number of scaffolds. Completeness is the estimate of the fraction of genes present in the genome with respect to the expected gene content. Completeness is determined based on universally distributed orthologs. We used Benchmarking Universal Single-Copy Orthologs (BUSCO) (Simão et al., 2015) to estimate genome completeness. BUSCO estimates complete single-copy, duplicated, fragmented, and missing genes in the data. The number of scaffolds shows how fragmented the assembly is, with a larger number indicating a more fragmented assembly.

Using Funannotate v1.8.9 on the resulting assemblies to estimate the number of genes and proteins (Palmer and Stajich, 2019). Funannotate implements the algorithm evidence modeler for gene prediction, which uses several different gene prediction inputs (from Augustus, snap, GlimmerHMM, CodingQuarry, and GeneMark-ES/ET) (Borodovsky and Lomsadze, 2011). In the functional annotation step, Funannotate identifies Pfam domains, carbohydrate-active enzymes, secreted proteins, proteases (via MEROPS), BUSCO groups, gene ontology, InterPro terms, and fungal transcription factors.

### Biosynthetic gene cluster prediction and clustering: AntiSMASH

Biosynthetic gene clusters were predicted using antiSMASH (Antibiotics and SM Analysis Shell, v6.0), with scripts implemented in the Funannotate pipeline (Blin et al., 2019; Palmer and Stajich, 2019). We tested if a smaller genome size was correlated with a smaller number of BGCs. A correlation coefficient near zero indicates no correlation and a coefficient close to 1 indicates a positive correlation.

### Biosynthetic gene cluster clustering into BiG-FAM gene cluster families

The homologous BGCs present in the *Umbilicaria* genomes were grouped into GCFs using BiG-FAM, which clusters structurally- and functionally-related BGCs, and identifies the structurally most divergent BGCs by comparing the query BGCs with the 1,225,071 BGCs in the BiG-FAM database. The 1,225,071 BGCs in BiG-FAM are clustered into 29,955 GCFs based on similar domain architectures. A GCF comprises closely related BGCs, potentially encoding the same or very similar compounds. By enabling such clustering, BiG-FAM establishes the degree of similarity of BGCs of a query taxon to currently known (functionally pre-characterized) fungal and bacterial BGCs. The antiSMASH job ID of each *Umbilicaria* species was used as input for BiG-FAM analysis.

### Quantification of biosynthetic gene cluster diversity and species-specific biosynthetic gene clusters in *Umbilicaria*: BiG-SLiCE

We used BiG-SLiCE (Kautsar et al., 2021b) to identify the most unique or species-specific BGCs within *Umbilicaria*. BiG-SLiCE 1.1.0. is a networking-based tool that assesses relationships of BGCs in the dataset (i.e., *Umbilicaria* BGCs in our study) and estimates their distance within the dataset to identity unique, species-specific BGCs. The resulting distance (*d*) indicates how closely a given BGC is related to the other BGCs. BiG-SLiCE was run on the *Umbilicaria* BGC dataset (i.e., 217 BGCs from nine *Umbilicaria* spp.) using three different thresholds (400, 900, and 1800).

## Results

### Overview of biosynthetic gene clusters in the *Umbilicaria* genomes

**Figure 1:**
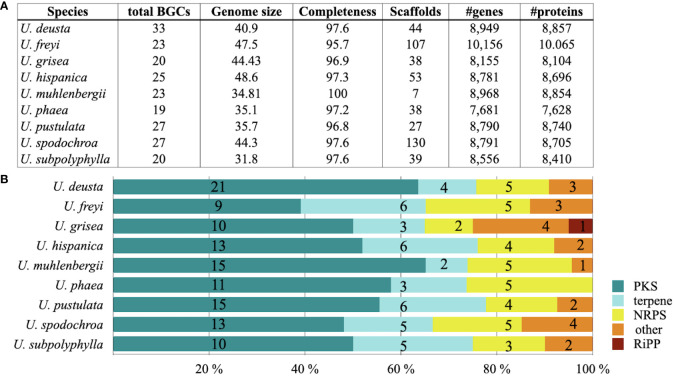
Genome quality metrics and diversity of biosynthetic genes in nine species of *Umbilicaria*. **(A)** Genome metrics, including the total number of biosynthetic gene clusters (BGCs) as predicted by antiSMASH, and the number of genes and proteins estimated by InterProScan and SignalP, as implemented in the Funannotate pipeline. **(B)** Diversity of BGCs associated with major natural product categories, indicated as percentages (colored bars) and absolute numbers (numbers on bars).

**Figure 2:**
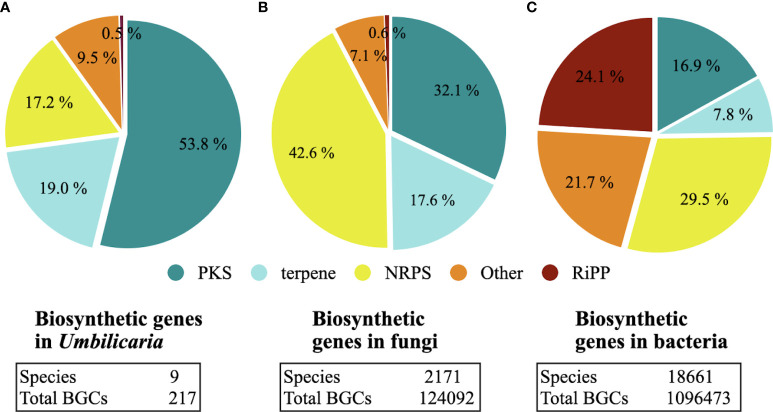
Biosynthetic gene clusters (BGCs) in **(A)** *Umbilicaria*, **(B)** the full fungal BGC dataset sensu Kautsar et al. 2021a, and **(C)** the full bacterial BGC dataset sensu Kautsar et al. 2021a. Polyketide synthases (PKSs) are the predominant class of BGCs in *Umbilicaria*, whereas in fungi and bacteria non-ribosomal peptide synthetases (NRPSs) are the most predominant BGC class. Although the number of publicly available lichen-forming fungal (LFF) genomes (> 50) is much smaller than the number of non-lichenized fungi (about 2,100), in all of the LFF genomes analyzed PKS clusters were the most common (see *Discussion* for details), suggesting that the predominance of PKSs, as observed here in the *Umbilicaria* dataset, is a common feature of LFF genomes.

*Umbilicaria* genomes contain 20–33 BGCs each, with the largest number of BGCs detected in *U. deusta* and the lowest in *U. phaea* (**Figure 1A**). We did not observe a correlation between genome size and number of BGCs (correlation coefficient = 0.10). *Umbilicaria* species contain an average of 13 PKS clusters and 4.2 NRPS clusters per species (**Figure 1B**), making a PKS to NRPS cluster proportion of 3.1. The most dominant classes of BGC in *Umbilicaria* are PKSs, which account for more than 50% of the total number of BGCs, followed by terpene clusters (about 20%) and NRPS clusters (about 15%) (**Figure 2A**). In contrast, NRPSs are the most dominant class among fungal and bacterial BGCs (**Figures 2B, C**), amounting to about 42% and 30%, respectively.

### Biosynthetic gene cluster clustering: BiG-FAM

**Figure 3:**
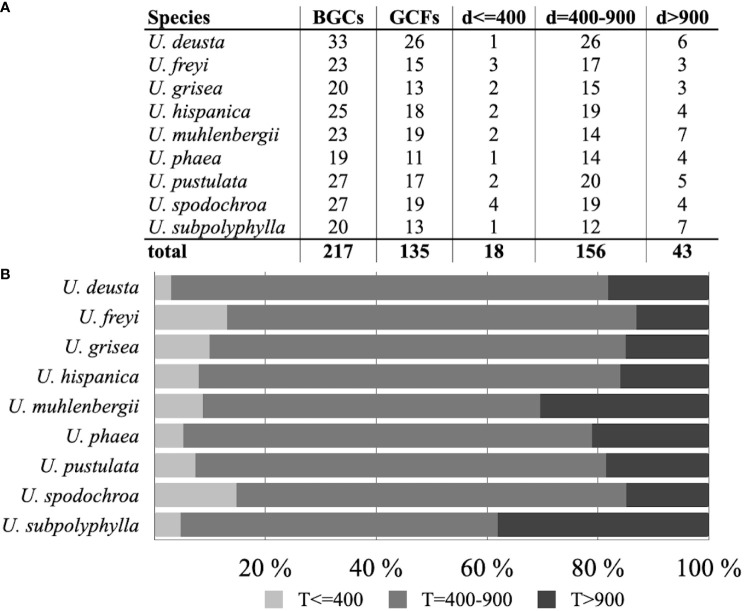
**(A)** Total biosynthetic gene clusters (BGCs) and gene cluster families (GCFs) as identified by BiG-FAM in Umbilicaria, along with the number of BGCs clustering into pre-characterized GCFs in BiG-FAM and their distance (d) groups. Distance is a measure of how closely a given BGC is related to other BGCs (*d* ≤ 400 suggests that the cluster codes for a structurally and functionally similar NP; *d* = 400–900 indicates that the BGC codes for a related but structurally and functionally divergent NP; and *d* > 900 suggests that the BGC potentially codes for a novel NP). **(B)** Bar plots representing the percentage of BGCs in each *Umbilicaria* species with *d* ≤ 400, *d* = 400–900, and *d* > 900. Only a small proportion of BGCs in each species could be grouped into a pre-characterized GCF in the BiG-FAM database (21,678 species, 1,225,071 BGCs, and 29,955 GCFs), whereas a large proportion of BGCs are only distantly related to the pre-characterized BGCs. Approximately 15%–30% of BGCs could not be grouped into BiG-FAM GCFs and, therefore, potentially code for structurally and functionally divergent NPs.

Of the total 217 BGCs found in nine *Umbilicaria* species, 18 (8%) BGCs obtained a BGC-to-GCFs pairing distance lower than 400, indicating that they potentially code for structurally very similar compounds known from the BGCs of their corresponging GCFs (**Figures 3A, B**). One hundred and fifty-six (72%) BGCs had a pairing distance of 400–900, suggesting that they share similar domain architectures with previously described BGCs in the BiG-FAM database. We describe the clusters belonging to above two groups as “associated product structurally known”. Forty-three (20%) BGCs had a pairing distance greater than 900, and are potentially BGCs encoding novel NPs (**Figure 3A**). We call these clusters “associated product putatively novel”. These BGCs belong to the classes terpenes (one BGC), NRPSs (12 BGCs), and PKSs (30 BGCs). The details of these BGCs and the sequence of the core gene are provided in **Supplementary Information S1**.

### Within-genus comparison of biosynthetic gene clusters: BiG-SLiCE

We identified species-specific BGCs within *Umbilicaria* using BiG-SLiCE. Out of 217 total BGCs, 159 (73%) grouped into 20 GCFs (*d* = 900), suggesting that they are similar clusters shared by multiple species, whereas 58 BCGs (27%) had *d* > 900, indicating that they were only distantly related to other BGCs in *Umbilicaria*. Each *Umbilicaria* species contains 4–10 (6.45%–16.13%) unique species-specific BGCs (**Supplementary Information 2A**). In *U. deusta*, we detected two BGCs (both with PKSs as the core gene) that were extremely divergent (*d* > 1,800) within the genus (**Supplementary Information 2B**). Of these BGCs, 15 were unique within *Umbilicaria*, as well divergent from the known BGCs present in the BiG-FAM database.

## Discussion

Lichens produce a large number of NPs, and they have even more BGCs (Meiser et al., 2017; Bertrand and Sorensen, 2018; Gerasimova et al., 2022). However, whether or not these BGCs encode hitherto unknown metabolically diverse chemical structures is not known. Here we quantify, for the first time, the proportion of BGCs linked to putatively novel NPs in a group of closely related LFF. The identification of 23 clusters that can encode putatively novel compounds can provide useful insights for novel drug leads.

**Figure 4:**
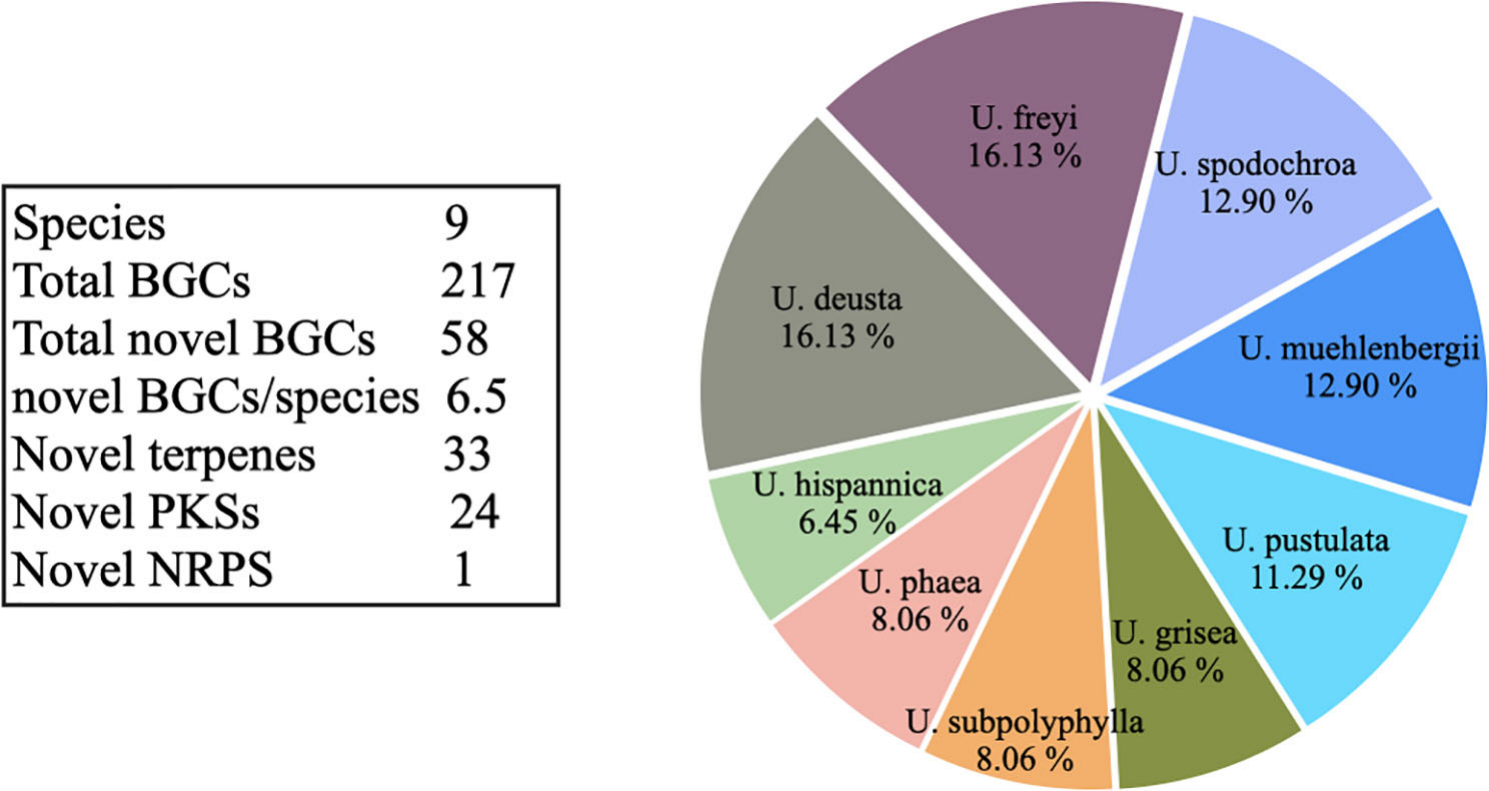
Pie chart depicting the contribution of each species to the overall novel *Umbilicaria* biosynthetic gene clusters (BGCs) (as identified by BiG-SLiCE, distance threshold T > 900) Each *Umbilicaria* species contains 4–10 unique, species-specific BGCs. *Umbilicaria freyi* and *U. deusta* contain the largest number of novel BGCs. The number of novel BGCs is slightly positively correlated to the number of clusters (*R* = 0.68). Of 58 unique BGCs (*T* > 900), 56.89% were terpene clusters and 41.37% were PKS clusters.

In this study, we mined the genomes of the *Umbilicaria* spp. to identify all BGCs (**Figure 1**), clustered the structurally noval BGCs and functionally similar BGCs into GCFs (**Figures 3A, B**), and identified gene clusters potentially coding for novel NPs (**Figure 4**; **Supplementary Information 1**). Using *Umbilicaria* spp. as a study system, we show that the LFF biosynthetic landscape is diverse from that of non-lichenized fungi and bacteria. The LFF biosynthetic landscape is particularly rich in PKSs (**Figure 2**), with a substantial portion of BGCs (about 28% in case of *Umbilicaria*) potentially coding for novel NPs (**Figures 3A, B**). To the best of our knowledge, this is the first investigation of this kind using state-of-the-art computational tools to determine the proportion of metabolic diversity in LFF potentially coding for novel compounds and to identify candidate genes as a source of drug leads to enable drug discovery efforts to be prioritized.

### Biosynthetic potential and biosynthetic gene cluster diversity of *Umbilicaria* spp.

Although only PKS-derived NPs are reported from *Umbilicaria* species (gyrophoric acid, umbilicaric acid, hiascic acid, etc.) (Posner et al., 1992; Davydov et al., 2017; Singh et al., 2021b), we found that the *Umbilicaria* BGC landscape is biosynthetically diverse and comprises three to five classes of NPs (**Figures 1A, B**). This is also the case for most other LFF; for instance, PKS-derived NPs are reported from *Bacidia* spp., *Cladonia* spp., *Endocarpon* spp., *Evernia prunastri*, *U. pustulata*, and *Pseudevernia furfuracea*, but all of them contain several PKS, NRPS, and terpene gene clusters (Calchera et al., 2019; Singh et al., 2021a; Singh et al., 2021b; Wang et al., 2021; Gerasimova et al., 2022). All the above-mentioned studies show that the biosynthetic potential of LFF vastly exceeds their detectable chemical diversity. On average, LFF may contain up to 30–40 BGCs, but the number of identified compounds per species is usually fewer than 10 (Calchera et al., 2019; Pizarro et al., 2020; Singh et al., 2021a). This could be because most of the clusters are silent and do not synthesize the NP, or it could be simply because of the failure to detect the NP. Bioinformatic characterization of entire BGC landscape followed by identification of most distinct BGCs provides a way to estimate the novelty of all BGCs, including the unexpressed and silent ones.

### Biosynthetic gene cluster diversity of non-lichenized fungi compared with bacteria and non-lichenized fungi

We identified five classes of BGCs in the *Umbilicaria* genomes. PKSs were the most dominant class, accounting for about 50% of BGCs, followed by terpenes (19%) and NRPSs (14%) (**Figures 1**, **2A**). BGCs, including PKS, typically make up the majority of BGCs in LFF, for instance about 60% in *E. prunastri*, 61% in *P. furfuracea*, 65% in *Cladonia* spp., 58% in *E. pusillum*, 46% in *Lobaria pulmonaria*, and 63% in *Ramalina peruviana* (Calchera et al., 2019; Kim et al., 2021; Singh et al., 2021a; Singh et al., 2021b).

Robey et al. (2021) identified 36,399 BGCs in 1,037 fungal genomes, which suggests that the average number of BGCs in a non-lichenized fungal genome is 35. This is lower than what has been reported from bacteria, with Liu et al. (2022) reporting 170,685 BGCs from 5,666 genomes (i.e., an average of 30 BCGs per genome). *Umbilicaria* species have, on average, 24 BGCs, which is lower than the average number of BGCs present in non-lichenized fungi and bacteria. However, *Umbilicaria* species, in general, are chemically not particularly diverse (Singh et al., 2022) and are, therefore, expected to have a smaller number of BGCs than other LFF.

Although the number of publicly accessible, good-quality genomes is somewhat lower for LFF (< 25), than for bacteria and non-lichenized fungi, the data available [nine *Umbilicaria* spp. genomes (Singh et al., 2022) plus nine other publicly available lichen genomes] suggest that the predominance of PKSs is a common feature of BGCs in LFF, accounting for more than 50% of the total number of BGCs. In contrast, NRPSs are the most prevalent BGC class in bacteria and non-lichenized fungi, accounting for about 30% and 42% of BGCs, respectively, followed by the PKSs (**Figures 2B, C**). This suggests that the biosynthetic potential of LFF is unique especially with respect to PKS diversity. In this regard, a recent study suggested that, although bacteria and fungi may share a few NPs, they do not have an overlapping chemical space and, instead, have distinct biosynthetic potential (Robey et al., 2021). LFF, having a distinct BGC landscape, present a complementary source of NPs with promising medicinally relevant biosynthetic properties.

### *Umbilicaria* biosynthetic gene cluster: gene cluster families and novel natural products

Gene cluster families are the groups of BGCs that encode the same or very similar molecules. A total of 217 BGCs from nine *Umbilicaria* species were clustered into 135 unique GCFs. (**Figure 3A**). This suggests that *Umbilicaria* spp. are potentially capable of synthesizing many structurally and functionally different NPs, although in nature only one compound class is typically detected (depsides, coded by the BGCs with PKS as the core gene.

Only a small fraction (8%) of *Umbilicaria* BGCs could be clustered with the pre-characterized BGCs (**Figures 3A, B**). About 71% of the BGCs were clustered to BiG-FAM GCFs with distance greater than 400–900, indicating that they were only distantly related in structure and function (**Figures 3A, B**). These BGCs are also interesting candidates to be investigated for their biosynthetic properties, as even a minor difference in the cluster and the chemistry of the final metabolites could cause a crucial difference in bioactivity related to function and the pharmacological potential of the product (Lautié et al., 2020).

Approximately 21% of BGCs were highly divergent (*d* > 900) and are novel, potentially coding for structurally and functionally unique NPs, and could, therefore, be an interesting target for NP-based drug discovery (**Figure 3B**). The strikingly large number of novel BGCs in a single fungal genus adds to the mounting evidence that non-model and understudied taxa are an enormous, untapped source of novel NPs.

Genome mining for large genomic regions, such as fungal BGCs, works best when the genomes under study are complete and contiguous, as well as reliably annotated. Many publicly available LFF genomes do not fulfill these criteria, thus preventing a taxonomically broad study of biosynthetic novelty encoded in the genomes of LFF. We were surprised that even a “chemically boring” lichen taxon, such as the genus *Umbilicaria*, harbored 43 BGCs putatively encoding a diverse range of previously unknown NPs. This leads us to speculate that chemically more diverse taxa, for example, Lecanorales or Pertusariales, each of which includes hundreds of species, are even richer sources of BGCs with novel functions and of compounds with potential novel pharmaceutical applications.

### Unique biosynthetic gene clusters within *Umbilicaria* spp.: BiG-SLiCE

Biosynthetic gene clusters that are unique to one species are candidates for interesting NPs (Navarro-Muñoz et al., 2020; Kautsar et al., 2021b; Robey et al., 2021). On average, each *Umbilicaria* species contains seven species-specific BGCs. *U. deusta* and *U. freyi* have the greatest number of novel BCGs, whereas *U. hispanica* contains the fewest (**Figure 4**). This suggests that even closely-related species (i.e., species within a single genus) contain diverse biosynthetic potential. Species- or strain-specific biosynthetic potential has already been demonstrated for LFF, for example in *U. pustulata* (Singh et al., 2021b) and *P. furfuracea* (Singh et al., 2021a), and it is rather common among fungi (Alam et al., 2021; Robey et al., 2021; Singh et al., 2021b). For instance, the majority (57%) of the BGCs in *Streptomyces* are strain specific (Choudoir et al., 2018). The unique BGCs within *Umbilicaria* belong to the BGC classes PKSs, terpenes, and NRPSs, as well as to the indoles (**Supplementary Information S2**). Notably, of these classes, only PKS-derived NPs have been well studied in LFF. Several studies have shown PKS-derived NPs to have diverse pharmacological properties (Manojlović et al., 2012; Cardile et al., 2017; Ingelfinger et al., 2020).

Two PKSs obtained a pairing distance greater than 1800. These PKSs were the most divergent (**Supplementary Information S2**) within *Umbilicaria* and are “orphan (i.e., clusters for which corresponding metabolite cannot be predicted). Recently, several orphan clusters have been activated to synthesize a compound with useful pharmacological properties; for example, the antibiotic holomycin gene cluster from the marine bacterium *Photobacterium galatheae* (Mattern et al., 2015; Shi et al., 2019; Ziko et al., 2019; Buijs et al., 2020). The novel and orphan clusters reported in this study are potentially interesting source of molecules with unique pharmacological properties and may novel serve as drug leads.

About 17% of fungal BGCs, 8% of bacterial BGCs, and 19% of LFF BGCs are terpenes (**Figure 2**). Terpenes are pharmaceutically extremely versatile, having antimicrobial, anti-inflammatory, neurodegenerative, and cytotoxic properties (Jaeger and Cuny, 2016; Cox-Georgian et al., 2019; Guimarães et al., 2019; Jiang et al., 2020; Yang et al., 2020; Del Prado-Audelo et al., 2021). Among the most common plant-derived terpenes and terpenoids are curcumin and eucalyptus oil. Although several studies have reported the pharmacological properties of fungal terpenes, such studies on LFF-derived terpenes are lacking, even though LFF genomes contain higher number of terpenes. In this study, we also report structurally and functionally unique terpenes as promising candidates to be investigated for their pharmaceutical potential.

## Data availability statement

Publicly available datasets were analyzed in this study. These data can be found here: National Center for Biotechnology Information (NCBI) BioProject, https://www.ncbi.nlm.nih.gov/bioproject/, with accession: PRJNA820300 and Figshare, https://figshare.com/, with accession: 10.6084/m9.figshare.19625997. The datasets supporting the conclusions of this article are available in the GenBank repository, accession PRJNA820300, under the accession numbers JALILQ000000000-JALILY000000000. The lichen samples of the corresponding Umbilicaria spp. are available as BioSamples SAMN27294873–SAMN27294881 and the mycobiont samples are available as BioSamples SAMN26992773–SAMN26992781. The antiSMASH files of Umbilicaria spp. are available at Figshare (DOI: 10.6084/m9.figshare.19625997).

## Author contributions

GS analyzed and interpreted the data, generated the figures and tables, and wrote the manuscript. FG analyzed the data and assisted with the bioinformatic parts of the study. IS interpreted the data, co-prepared the figures, and co-wrote the manuscript. All authors read and approved the final manuscript.

## Funding

This research was funded by LOEWE-Centre TBG, funded by the Hessen State Ministry of Higher Education, Research and the Arts (HMWK).

## Acknowledgments

We thank Professor Marnix Medema and Dr Satria Kautsar for their support with the BiG-SLiCE program.

## Conflict of interest

The authors declare that the research was conducted in the absence of any commercial or financial relationships that could be construed as a potential conflict of interest.

## Publisher’s note

All claims expressed in this article are solely those of the authors and do not necessarily represent those of their affiliated organizations, or those of the publisher, the editors and the reviewers. Any product that may be evaluated in this article, or claim that may be made by its manufacturer, is not guaranteed or endorsed by the publisher.

## Supplementary material

Supplementary Information S1 Most divergent BGCs in *Umbilicaria*, as identified by BiG-FAM, along with the cluster information and sequence.

Supplementary Information S2 Most distantly related BGCs within *Umbilicaria*, as identified by BiG-SLiCE, along with the cluster information.
